# Delta Radiomic Analysis of Mesorectum to Predict Treatment Response and Prognosis in Locally Advanced Rectal Cancer

**DOI:** 10.3390/cancers15123082

**Published:** 2023-06-07

**Authors:** Giuditta Chiloiro, Davide Cusumano, Angela Romano, Luca Boldrini, Giuseppe Nicolì, Claudio Votta, Huong Elena Tran, Brunella Barbaro, Davide Carano, Vincenzo Valentini, Maria Antonietta Gambacorta

**Affiliations:** 1 Fondazione Policlinico Universitario “A. Gemelli” IRCCS, Largo Agostino Gemelli 8, 00168 Rome, Italy; giuditta.chiloiro@policlinicogemelli.it (G.C.); luca.boldrini@policlinicogemelli.it (L.B.); giuseppe.nicoli@guest.policlinicogemelli.it (G.N.); claudio.votta@guest.policlinicogemelli.it (C.V.); huongelena.tran@policlinicogemelli.it (H.E.T.); brunella.barbaro@policlinicogemelli.it (B.B.); davide.carano@guest.policlinicogemelli.it (D.C.); vincenzo.valentini@policlinicogemelli.it (V.V.); mariaantonietta.gambacorta@policlinicogemelli.it (M.A.G.); 2 Mater Olbia Hospital, Strada Statale Orientale Sarda 125, 07026 Olbia, Italy; davide.cusumano@materolbia.com

**Keywords:** rectal cancer, radiomics, mesorectal fact signatures, predictive models, high-risk factors, early regression index

## Abstract

**Simple Summary:**

Early prediction of response to cancer therapies is critical for treatment personalisation. In patients with locally advanced rectal cancer (LARC) undergoing neoadjuvant chemoradiation therapy (nCRT), delta radiomics applied to mesorectal features could potentially lead to the development of predictive models of treatment response. Pre- and post-treatment MRIs of patients treated at a single institution were analysed. Radiomic features of the mesorectum and GTV were extracted and predictive models of pathological complete response (pCR) and two-year disease-free survival (2yDFS) were developed. In 203 patients with LARC, a total of 565 variables were evaluated; the best performing 2yDFS prediction model was based on one GTV and two mesorectal features with an AUC of 0.79 in the training set and 0.70 in the validation set. The mesorectum may contain important radiomics information for predicting response to nCRT treatment in LARC patients.

**Abstract:**

Background: The aim of this study is to evaluate the delta radiomics approach based on mesorectal radiomic features to develop a model for predicting pathological complete response (pCR) and 2-year disease-free survival (2yDFS) in locally advanced rectal cancer (LARC) patients undergoing neoadjuvant chemoradiotherapy (nCRT). Methods: Pre- and post-nCRT MRIs of LARC patients treated at a single institution from May 2008 to November 2016 were retrospectively collected. Radiomic features were extracted from the GTV and mesorectum. The Wilcoxon–Mann–Whitney test and area under the receiver operating characteristic curve (AUC) were used to evaluate the performance of the features in predicting pCR and 2yDFS. Results: Out of 203 LARC patients, a total of 565 variables were evaluated. The best performing pCR prediction model was based on two GTV features with an AUC of 0.80 in the training set and 0.69 in the validation set. The best performing 2yDFS prediction model was based on one GTV and two mesorectal features with an AUC of 0.79 in the training set and 0.70 in the validation set. Conclusions: The results of this study suggest a possible role for delta radiomics based on mesorectal features in the prediction of 2yDFS in patients with LARC.

## 1. Introduction

Rectal cancer is the third most common neoplasm, mainly affecting people between the ages of 40 and 50. The risk of developing it is associated with environmental factors such as type II diabetes, increased body mass index and obesity, but chronic inflammatory bowel disease, smoking and alcohol consumption can also increase the risk [1,2].

After accurate staging of the disease in a multidisciplinary setting, with digital rectal examination, endoscopic examination with histological confirmation and execution of pelvic MRI with MDC and total body CT scan, it is possible to identify patients with locally advanced rectal cancer (LARC) [1]. Standard treatment for LARC is multimodal, with neoadjuvant chemoradiotherapy (nCRT) and/or radiotherapy (RT) followed by total mesorectal excision (TME) as the cornerstones [3,4]. The integration and further evolution of these approaches into the treatment workflow has made it possible to increase both locoregional control (LC) and overall survival (OS) rates, which are currently the most reliable endpoints of treatment efficacy.

In recent years, there has been a growing interest in identifying biomarkers that can predict early response to therapy. The aim is to further personalise treatment strategies and thus increase treatment efficacy [5].

Several studies have shown that complete pathological response (pCR), achieved in approximately 15–30% of patients following nCRT [6,7], is a strong surrogate for overall survival (OS) and is the most important early endpoint for assessing overall treatment efficacy [5].

In recent years, various strategies have been employed to increase pCR and thus reduce the risk of local recurrence (LR), such as RT dose escalation [8], chemotherapy (CHT) intensification [9] and increasing the time interval before surgery [6]. Although some of these approaches have shown an increase in pCR rates, no significant improvement in disease-free survival (DFS) has been reported in these and related trials [10,11].

More than pCR, early development of metastases is considered a strong predictor of OS probability and may identify biologically aggressive tumours [12].

Recently, several factors have been proposed to identify more aggressive biological tumour behaviour. These include extramural vascular invasion (EMVI), involvement of the mesorectal fascia (MRF), the presence of pathological extra-mesorectal lymph nodes and tumour deposits [13,14].

The surrounding tumour microenvironment also plays a key role: indeed, the tumour immune microenvironment (TIME) after radiochemotherapy is known to exhibit specific immunological characteristics [15], although to date no molecular biomarker has been identified that predicts response to nCRT and is considered sufficiently robust to be used in clinical practice [16].

In recent years, the role of radiomics has emerged and become relevant in rectal cancer, as various experiences have shown that the combination of clinical and radiomic features can accurately predict the biological behaviour of the tumour and the main survival outcomes (pCR, OS and DFS) [17,18,19,20,21,22,23].

Phenotypic expression of gross tumour volume (GTV) is the most commonly used approach in radiomics for rectal cancer, without consideration of TIME.

Recently, several predictive models for LARC have been proposed using radiomic and delta-radiomic approaches using GTV as a region of interest (ROI): interesting results have been reported for pCR, DFS and distant metastasis-free survival (DMFS) [18,19,20,23,24,25,26].

Beyond radiomic models, simple image-based biomarkers, easy to calculate and based on robust radiobiological theories, have been proposed for rectal cancer: it is the case of the early regression index (ERI), a morphological parameter based on measuring the GTV volume at the time of simulation and at the time of the 10th fraction of nCRT, which showed remarkable performance in predicting pCR and DFS [27,28].

This index has also been proposed and validated as a predictor of pCR in magnetic resonance guided RT (MRgRT) for LARC [29] and more generally has shown to be a promising response biomarker in other pelvic malignancies, such as cervical cancer [30].

The potential of TIME for response prediction and patient stratification has been analysed in few experiences in rectal cancer [31,32].

Such experience, focusing on radiomic analysis of the mesorectum, has shown that mesorectal fat may contain important prognostic information in patients with LARC.

Indeed, features extracted from mesorectal fat have been shown to predict pCR and, in combination with primary tumour features, also tumour regression grade (TRG) and neoadjuvant rectal (NAR) score, which provides important information on the efficacy of neoadjuvant treatments [31,32].

Based on these findings, the aim of this study was to investigate the predictive performance of delta radiomics features extracted from mesorectum and rectal tumours using pre- and post-nCRT magnetic resonance imaging (MRI) in predicting pCR and 2-year DFS (2yDFS).

## 2. Materials and Methods

### 2.1. Study Population

A retrospective analysis of data from LARC patients treated at our institution between May 2008 and November 2016 was performed.

Inclusion criteria were: (a) patients older than 18 years old; (b) pathologically proven rectal adenocarcinoma; (c) nCRT followed by surgery; (d) both pre-treatment (staging) and post-treatment (re-evaluation) MRI performed in our institution; (e) follow-up information of at least 2 years from the date of surgery.

All patients underwent RT treatment with a total dose of 45 Gy (1.8 Gy/day) on the whole mesorectum and selected lymphatic drainage stations, according to disease stage [33]. The total dose to the tumour and the corresponding mesorectum was 50.4 Gy delivered by a sequential boost of 5.4 Gy/day over 3 fractions or 55 Gy delivered by a sequential or simultaneous integrated boost (SIB) for a daily dose of 2 Gy and 2.2 Gy, respectively, over a five weeks treatment period [33].

The concurrent CHT regimens used were CapOx (60 mg/m^2^ of iv oxaliplatin on day 1 plus 1300 mg/day/m^2^ of oral capecitabine, days 1 to 7, q7), chronomodulate oral capecitabine (1650 mg/m^2^ days 1 to 7 or 1 to 5 q7 during radiotherapy) [34] or 5-fluorouracil in continuous infusion (225 mg/m^2^/day from day 1 to 7 q7 during RT), depending on clinical stage and patient compliance.

Surgery was performed 8–12 weeks after the end of nCRT following clinical and radiological restaging.

Pathological staging was carried out according to the pTNM classification [35] and tumour response was assessed by tumour regression grade (TRG) according to the Mandard’s classification [36]. The pathological response was assessed for each patient, and pCR was defined as the absence of viable tumour cells in the pathological specimen (ypT0ypN0) after nCRT.

Adjuvant CHT was administered in selected patients, according to tumour characteristics.

All patients enrolled in the trial were followed up according to standardised institutional assessment protocols.

For long-term endpoints, 2yDFS was defined as the absence of distant metastasis or local recurrence within 2 years from the date of surgery.

### 2.2. Image Analysis

Pre- and post-treatment MR images were acquired using a 1.5 T MR scanner (Horizon Advantage, GE Medical Systems, Milwaukee, WI, USA) with pelvic surface phased-array coils. Scanning was performed in the supine position for all patients. In order to reduce image artefacts, an ultrasound gel enema was administered to distend the rectal lumen and limit luminal air. An intramuscular antiperistalsis agent, hyoscine-N-butyl bromide (20 mg), was also administered. All MR scans were acquired following an internal standard protocol for rectal cancer consisting in a T2-weighted fast spin echo (FSE) sequence acquired in axial, coronal and sagittal planes. In addition, an oblique T2-weighted FSE 3D high-resolution images was acquired in a plane orthogonal to the tumour. Technical parameters for the two MR sequences are reported in Table S1 of Supplementary Materials.

At the post-treatment MRI examination, by integrating DWI and high-resolution T2-weighted MR images, a decrease in signal intensity when compared with the pre-treatment examination was considered to represent response with fibrosis.

The pre- and post-treatment DICOM MR images of each patient were exported from the institutional PACS system and uploaded on a RT delineation console (Eclipse, Varian Medical System™, Palo Alto, CA, USA).

**Figure 1:**
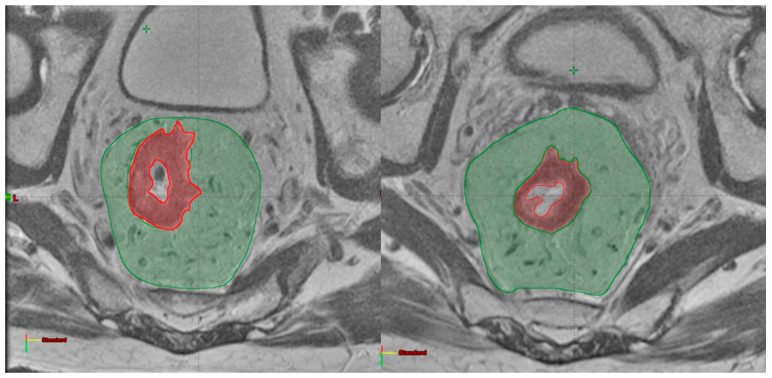
GTV (red) and mesorectum (green) delineated on pre-treatment (**left**) and post-treatment (**right**) MRI on axial plans.

A radiation oncologist delineated two regions of interest: the GTV and the corresponding mesorectum, as shown in Figure 1. All the delineations were performed in agreement with a second radiation oncologist with expertise in rectal cancer. Delineation was blinded to histopathological results.

The GTV was delineated on the axial oblique T2-weighted images, including the entire tumour and excluding the lumen, stools and air bubbles. The hypointense area on T2-weighted pre-treatment MR corresponding to the primary tumour was contoured on post-treatment MRI in the case of an apparent complete response on MRI [37]. The mesorectal volume, represented by the mesorectal fat bounded by the mesorectal fascia, was obtained by boolean subtraction of the GTV from the mesorectum.

### 2.3. Radiomic Analysis

Moddicom, a dedicated platform for radiomics analysis, was used to analyse the DICOM files containing the pre- and post-treatment MR images and the RT structure files containing the GTVs and mesorectal structures of all cases [38,39].

From the GTV and mesorectal ROIs of each pre- and post-treatment MRI, different families of radiomic features (statistical, morphological, textural) were extracted. Delta radiomic features were calculated as the ratio of features obtained before and after treatment.

*ERI* was calculated for GTV and mesorectum considering the following formula:

(1) $$ERI=-ln\left[{\left(1-\left(\frac{V_{mid}}{V_{pre}}\right)\right)}^{V_{pre}}\right]$$

where *V_pre_* is the GTV volume measured at the time of simulation and *V_mid_* is the volume measured at the end of treatment [27,40].

The dataset was randomly split into 66% for the training set and 33% for the validation, according to the transparent reporting of a multivariable prediction model for individual prognosis or diagnosis (TRIPOD) [41].

In addition to radiomic features, the following clinical variables were considered: age, gender, cT stage, cN stage, presence of extra-mesorectal lymph nodes, mesorectal fascial involvement, clinical stage, RT dose prescribed to the GTV, type of neoadjuvant CHT, number of cycles of neoadjuvant CHT, number of cycles of adjuvant CHT and neoadjuvant CHT, downstaging stage, downstaging TNM and response to the nCRT (pCR).

The Wilcoxon–Mann–Whitney (WMW) test was performed to identify the ability of each parameter in predicting pCR and 2yDFS, at the univariate analysis.

The Pearson correlation coefficient (PCC) was used to estimate the correlation between parameters that showed statistical significance (*p* < 0.05) in the univariate analysis.

For each outcome defined, different logistic regression models were developed using the features showing statistical significance at the univariate analysis.

Being a comparative study among predictive models based on different ROIs, logistic regression was preferred to machine learning algorithms to maintain higher level of model interpretability, focusing only on linear relationships between variables and outcome.

The Delong test was used to compare the receiver operating characteristic (ROC) curves of the different models tested [42].

The performance of these models was assessed using the ROC curve. The 95% confidence interval for the area under the ROC curve (AUC) was calculated using 2000 stratified bootstrap replicates [43].

The performance of the different prediction models was therefore calculated in terms of specificity, sensitivity and overall accuracy. Such parameters have been evaluated at the best cut-off threshold, obtained maximizing the Youden Index, calculated as reported in similar experiences dealing this topic [29,30].

## 3. Results

The study included 203 consecutive LARC patients (65% men, 35% women). The median age was 65 years (range 26–83 years).

Both clinical and pathological complete response was reached in 54 patients (26.6%).

At a median follow-up of 95 months (range 60–156), the 2yDFS was 84.3% with a 5y-OS of 83.7%.

Details of the patients’ clinical and treatment parameters are summarised in Table 1.

A total of 546 radiomic features (273 mesorectal features, 273 GTV features) were calculated and evaluated for the two outcomes, together with 19 clinical variables.

Tables S2 and S3 in the Supplementary Materials showed the parameters with higher statistical significance at the univariate analysis for pCR and 2yDFS prediction.

The best-performing pCR prediction model was based on the combination of ERI and grey level non uniformity based on run length matrix (rlm.glnu) calculated on the GTV delineated on the MRI acquired post nCRT: the model coefficients are reported in the Supplementary Materials. This model showed an AUC of 0.80 (95% confidence interval (95%CI) ranging from 0.71 to 0.89) in the training set and 0.69 (95% CI of 0.56–0.81) in the validation set. Nevertheless, this model did not differ significantly (*p* = 0.20 at the Delong test) from the model based on the GTV_ERI alone (AUC of 0.79, with a range of 0.68–0.78).

**Figure 2:**
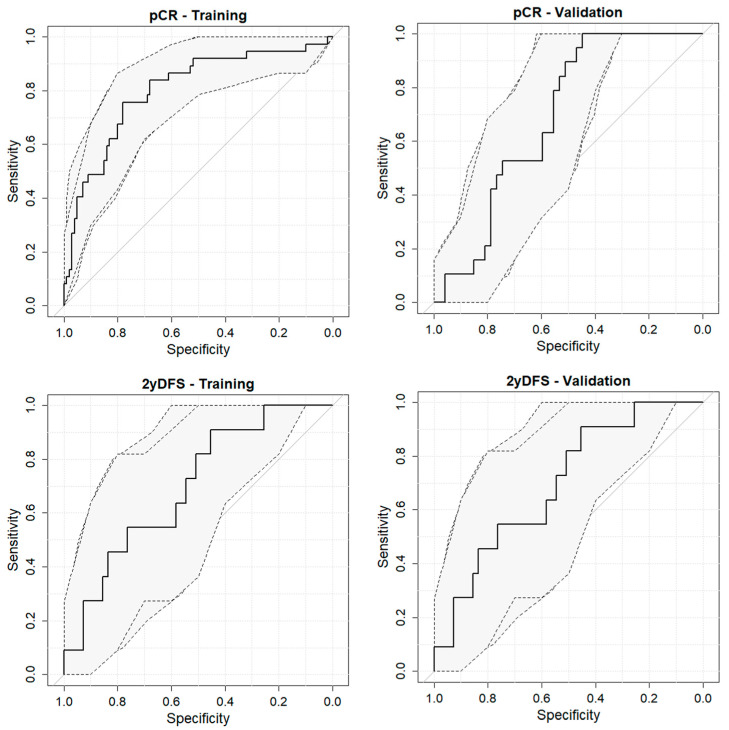
ROC (receiver operating characteristic) curves of the models elaborated for pCR (pathological complete response, top line) and 2yDFS (disease free survival at 2 years, bottom line) for training (**left**) and validation set (**right**), respectively.

Regarding 2yDFS prediction, the best-performing prediction model was based on three radiomic features: Variation in dissimilarity of GTV during treatment, energy calculated within the mesorectum of the MRI pre-nCRT and kurtosis of the mesorectum calculated on the post-nCRT MRI. Such a model, whose coefficients are reported in the Supplementary Materials, showed an AUC of 0.79 (95% CI equal to 0.69–0.90) in the training set and 0.70 (95% CI of 0.53–0.86) in the validation set (Figure 2).

Sensitivity and specificity values for the different outcomes are summarized in Table 2.

## 4. Discussion

Our study evaluated radiomic and delta radiomic features in 203 LARC patients undergoing nCRT followed by surgery. Specifically, a total of 546 features were extracted from both the GTV and the mesorectal fat.

The developed model is able to discriminate patients undergoing pCR with an AUC of 0.79 by incorporating features based on the ERI GTV, with no increase in discriminatory power when the feature rlm.glnu calculated on the GTV delineated on post-nCRT MRI is added. This result further confirms the discriminatory power of the ERI index to predict pCR, a bioimaging marker that can be easily calculated and implemented in a therapeutic workflow [28,29]. The pCR prediction line of investigation is in accordance with recent studies that have focused on the development of radiomic models capable of predicting tumour behaviour in terms of response or non-response to treatment from features derived from GTV analysis [24].

However, it is now widely accepted that pCR alone provides only partial information about the tumour behaviour: Valentini et al. demonstrated that 2yDFS is a better surrogate than pCR for predicting OS in a pooled analysis of 2795 rectal cancer patients who received long-course RT with or without concurrent and/or adjuvant CHT [43].

In the last few years, a correlation has been shown between the variation in radiological characteristics on MRI before and after nCRT and the two-year distant metastatis (2y-DM) rate in LARC patients [25]. This result was also investigated during MRgRT treatment, showing that the best performance is achieved with a pure radiomic model. In particular, the change in area/surface ratio of the GTV at 54 Gy and in the simulation predicted 2y DFS with an AUC of 0.92 [44].

However, recent studies have shown that there are other prognostic factors for predicting 2yDFS, such as tumoral deposits, EMVI, MRF involvement and positive nodes [14,45,46], that are located in the mesorectum.

The role of these features and their prognostic value was already deeply analysed from a clinical and, recently, from a radiological point of view: the preoperative identification of prognostic features allows the stratification of patients into different prognostic groups based on the assessment of the depth of extramural spread, the relationship of the tumour edge with the MRF and the presence of EMVI. The combination of these prognostic features predict an increased risk of local recurrence and/or metastatic disease [47].

Recently, starting from these evidences, several MRI-based radiomics models for preoperative prediction of EMVI [48], lymph node metastasis and tumour deposits [48] were elaborated.

In particular, Yan et al. [47] validated two nomograms based on the radiomics characteristics of the pre-treatment MR images of 139 rectal cancer patients for the prediction of lymph node metastases and tumour deposits, while Yu et al. [49], analysing the pre-treatment and DCE MR images of 106 LARC patients, demonstrated the better diagnostic accuracy of a radiomics nomogram that incorporates the radiomics score, histopathological grade and T stage than the DCE-MRI parameters for the prediction of EMVI.

Based on these assumptions, we hypothesized that the micro- and macro-environment of the mesorectum might contain relevant information regarding response to therapy and, in particular, the risk of developing a recurrence. The model obtained from our study to predict 2yDFS combines GTV and mesorectum radiomic features and exhibits an AUC of 0.76 in training and 0.7 in validation, respectively.

Our results show that the mesorectal fat is potentially rich in predictive information that may approach the predictive information of the tumour itself. A recent study of Shaish et al. [31] analysed the pre-treatment MRIs of 132 LARC patients and obtained the best classification performance for the prediction of pCR based on mesorectal features alone and based on both mesorectal and primary tumour features for the prediction of TRG and NAR scores. The authors highlighted the importance in the power of prediction and information contained in mesorectal fat, despite the heterogeneity of the imaging data.

This evidence is also confirmed by Jayaprakasam et al. [32], in a retrospective study including mesorectal segmentations on pre-treatment MRIs of 236 LARC patients, obtaining a model with an AUC of 0.86 in pCR prediction, AUC of 0.79 for LC and 0.87 for distant recurrence.

Compared to previous works, our study focused on the mesorectal radiomics features and their prognostic value when added to GTV radiomics features in terms of ability of predicting pCR and DFS, combining pre-, post- and delta features extracted from GTV and mesorectum both.

These results confirm that the mesorectum and the environment surrounding the tumour contain crucial information to better understand tumour behaviour and aggressiveness earlier. The information gained from delta-radiomics could be used in clinical practice to guide therapeutic decisions, for example by choosing to intensify treatment in patients who are more likely to develop disease recurrence, leading to improved patient outcomes. In addition, the mesorectum is an easily identifiable structure on MR images, which could be used in cases where the primary tumour is difficult to delineate. The rectum itself may have inherent anatomical and physiological variations that can introduce confounding factors when analysing radiomic features. By focusing on the mesorectum, which is relatively standardised in terms of anatomy, the potential influence of confounding factors related to the rectal wall or luminal contents can be minimised. This may improve the reproducibility and reliability of radiomic analysis.

However, the present study suffers from some limitations: firstly, it is a retrospective analysis with a wide accrual period; consequently, there is a lack of information about the others prognostic factors such as EMVI status, the presence of tumoral deposits, mucinous histotype and biochemical parameters. Added value could come from combining radiomic information with that derived from lymphocyte profiles or other blood parameters, which have shown predictive power for radiotherapy response in other settings [50].

Furthermore, the study lacks external validation with an independent cohort of patients. If confirmed on external cohorts, the predictive model here proposed could help to identify poor prognosis patients early and so design RT dose escalation or CHT intensification protocols in the framework of even more personalised treatments for LARC patients.

## 5. Conclusions

In conclusion, this study demonstrated the importance of including mesorectum in radiomic analysis, showing its important role in improving the predictive performance of radiomic models based only on GTV analysis, especially in the case of 2yDFS.

## Figures and Tables

**Table 1:** Patient characteristics.

	Number of Patients (%)
Median age (range)	65 (26–83)
Gender	
Male	132 (65)
Female	71 (35)
cT	
2	14 (6.9)
3	119 (58.6)
4	70 (34.4)
cN	
0	13 (6.4)
1–2	190 (93.6)
Stage	
II	13 (6.4)
III	190 (93.6)
Median RT dose [Gy] (range)	55 (50–59.4)
ypT0/in situ	58 (28.6)
1	3 (1.48)
2	60 (29.55)
3	76 (37.44)
4	6 (2.95)
ypN	
0	151 (74.38)
1–2	52 (25.62)
pCR/cCR	
Yes	54 (26.6)
No	149 (73.4)
2yDFS	
Yes	171 (84.3)
No	32 (16.7)

pCR: pathological complete response; cCR: clinical complete response; 2yDFS: 2 years disease free survival; RT: radiotherapy.

**Table 2:** Sensitivity and specificity values for pCR and 2yDFS.

Outcome	Dataset	Sensitivity	Specificity	AUC
pCR	Training	75.7	78.0	80.2 (71.4–88.9)
	Validation	100.0	44.7	68.6 (56.0–81.2)
2yDFS	Training	61.9	87.9	79.2 (68.7–89.6)
	Validation	90.9	45.5	69.6 (53.5–85.7)

## Data Availability

The data presented in this study are available on request from the corresponding author.

## Supplementary Materials

The following supporting information can be downloaded at: https://www.mdpi.com/article/10.3390/cancers15123082/s1, Table S1: Technical parameters for MRI acquisition; Table S2: Single features significant at the univariate analysis for pCR prediction; Table S3: Single features significant at the univariate analysis for 2yDFS prediction.
