# Supplementary material for: Delta Radiomic Analysis of Mesorectum to Predict Treatment Response and Prognosis in Locally Advanced Rectal Cancer
Source: Cancers (Basel). 2023 Jun 7;15(12):3082. doi: 10.3390/cancers15123082 (PMC10296157; doi:10.3390/cancers15123082)
Supplement: Supplementary file 1 [file cancers-15-03082-s001.zip › cancers-2398836-supplementary.pdf]

Table S1: Technical parameters for MRI acquisition.

Parameter	Training set
Magnetic Field Strength	1.5 T
FOV	18 cm
Repetition time	2500-5000 msec
Inversion time	100-110 msec
Echo train length	16-24
NEX	4
Pixel Spacing	0.703

Table S2: Single features significant at the univariate analysis for pCR prediction.

	features.name_def	all.pvalues	padj
1	post_GTV_F_morph.pca.flatness	0,000139	0,009036
2	post_GTV_maxFD	0,000181	0,010113
3	Tipo_chirurgia	0,000203	0,010113
4	post_GTV_F_morph.surface	0,000207	0,010113
5	post_GTV_F_stat.energy	0,000245	0,011083
6	pre_GTV_F_morph.av	0,000291	0,012192
7	post_GTV_F_rlm.rlnu	0,00036	0,014106
8	post_GTV_F_cm.auto.corr	0,000435	0,014863
9	cT.y	0,000448	0,014863
10	post_GTV_F_szm.zsnu	0,000456	0,014863
11	pre_GTV_F_morph.volume	0,0005	0,015449
12	post_GTV_F_cm.joint.entr	0,000574	0,016844
13	post_GTV_L_minor	0,000658	0,01756
14	post_GTV_F_cm.joint.avg	0,000688	0,01756
15	post_GTV_F_cm.sum.avg	0,000688	0,01756
16	CEA alla diagnosi	0,000975	0,022932
17	delta_GTV_F_cm.auto.corr	0,001025	0,022932
18	post_GTV_F_cm.sum.entr	0,001047	0,022932
19	post_GTV_F_cm.joint.max	0,00107	0,022932
20	pre_GTV_L_least	0,001094	0,022932
21	post_GTV_sdFD	0,001207	0,024427
23	post_GTV_medianFD	0,001379	0,026975
24	pre_GTV_F_cm.diff.entr	0,001445	0,027354

25	pre_GTV_F_morph.surface	0,001639	0,030061
26	CT_neoadiuvante.y	0,001697	0,030179
27	post_GTV_F_cm.energy	0,001781	0,03075
28	pre_GTV_F_morph.pca.flatness	0,001895	0,031783
29	ypT	1,94E-03	5,70E-01
30	pre_GTV_L_minor	0,001975	0,032199
31	pre_GTV_F_cm.inv.var	0,0021	0,033315
32	ypN	0,002255	0,034829
33	T4_invasione.y	0,002703	0,040683
34	stadio_rivalutazione_clinica	0,003223	0,04729
35	post_GTV_F_cm.sum.var	0,004113	0,056286
36	post_GTV_F_cm.clust.tend	0,004113	0,056286
37	pre_GTV_F_cm.diff.avg	0,004611	0,056286
38	pre_GTV_F_cm.dissimilarity	0,004611	0,056286
39	pre_GTV_F_cm.inv.diff	0,004611	0,056286
40	pre_GTV_F_cm.inv.diff.mom	0,004611	0,056286
41	delta_GTV_F_morph.pca.elongation	0,004611	0,056286
42	pre_GTV_F_cm.inv.diff.norm	0,004698	0,056286
43	post_GTV_F_cm.joint.var	0,004698	0,056286
44	post_Mesoretto_L_least	0,004879	0,056511
45	post_GTV_meanFD	0,00491	0,056511
46	pre_GTV_F_cm.inv.diff.mom.norm	0,005986	0,06757
47	post_GTV_F_cm.clust.prom	0,006324	0,070043
48	pre_Mesoretto_F_morph.pca.flatness	0,006559	0,0713
49	Stadio patologico	6,85E-03	1,34E+00
50	pre_GTV_F_cm.contrast	0,007578	0,080882
51	post_Mesoretto_F_morph.pca.flatness	0,008285	0,086841
52	post_GTV_F_szm.glnu	0,009704	0,099931
53	pre_GTV_F_cm.diff.var	0,010222	0,103455
54	post_GTV_F_cm.corr	0,011726	0,116663
55	pre_Mesoretto_L_least	0,011927	0,116685
56	pre_GTV_maxFD	0,012548	0,120574
57	post_GTV_F_stat.iqr	0,012735	0,120574
58	post_GTV_F_szm.z.entr	0,013646	0,127151
59	post_Mesoretto_F_morph.surface	0,014345	0,131571
60	post_Mesoretto_maxFD	0,014585	0,131712
61	delta_Mesoretto_minFD	0,016098	0,143172
62	post_GTV_F_rlm.glnu	0,01718	0,150522
63	delta_GTV_meanFD	0,018923	0,163351
64	pre_GTV_medianFD	0,020817	0,177091
65	pre_GTV_F_stat.energy	0,021483	0,177613
66	post_GTV_F_stat.rmad	0,021483	0,177613
67	delta_GTV_F_cm.diff.avg	0,021823	0,177918
68	post_GTV_F_cm.diff.entr	0,025098	0,199085
69	delta_Mesoretto_sdFD	0,025098	0,199085
70	pre_Mesoretto_F_morph.surface	0,025486	0,199472
71	pre_GTV_F_morph.pca.elongation	0,026279	0,201147

72	delta_GTV_F_morph.volume	0,027507	0,201147
73	delta_GTV_F_szm.zsnu.norm	0,027927	0,201147
74	post_GTV_F_szm.lzhge	0,028353	0,201147
75	delta_GTV_F_cm.info.corr.1	0,028353	0,201147
76	Hb alla diagnosi	0,028606	0,201147
77	delta_GTV_F_cm.sum.entr	0,028784	0,201147
78	delta_GTV_F_cm.clust.shade	0,028784	0,201147
79	delta_GTV_F_rlm.rlnu.norm	0,028784	0,201147
80	delta_GTV_F_cm.energy	0,031025	0,21247
81	delta_GTV_F_cm.clust.tend	0,03149	0,21247
82	delta_Mesoretto_medianFD	0,03149	0,21247
83	CT_adiuvante_precx	0,031868	0,212576
84	delta_GTV_F_stat.mean	0,033674	0,222096
85	ycN	0,035369	0,22215
86	post_GTV_F_morph.comp.1	0,035953	0,22215
87	post_GTV_F_morph.comp.2	0,035953	0,22215
88	post_GTV_F_morph.sph.dispr	0,035953	0,22215
89	post_GTV_F_morph.sphericity	0,035953	0,22215
90	post_GTV_F_morph.asphericity	0,035953	0,22215
91	post_GTV_F_rlm.rl.entr	0,037014	0,226323
92	pre_Mesoretto_F_cm.corr	0,038655	0,23392
93	delta_Mesoretto_F_stat.rms	0,039215	0,234889
94	post_GTV_F_cm.info.corr.1	0,043331	0,2493
95	pre_GTV_F_szm.glnu	0,043948	0,2493
96	post_GTV_F_cm.clust.shade	0,043948	0,2493
97	pre_GTV_meanFD	0,044572	0,2493
98	post_GTV_F_rlm.lrhge	0,044572	0,2493
99	post_Mesoretto_F_morph.volume	0,045203	0,2493
100	post_GTV_F_rlm.hgre	0,046488	0,2493
101	post_GTV_F_szm.hgze	0,046488	0,2493
102	delta_GTV_F_stat.rms	0,046488	0,2493
103	pre_GTV_L_major	0,047142	0,2493
104	post_GTV_F_stat.mad	0,047142	0,2493
105	delta_GTV_F_cm.joint.entr	0,047142	0,2493
106	delta_GTV_F_szm.sze	0,047142	0,2493
107	post_GTV_L_major	0,049834	0,258872
108	post_GTV_F_rlm.srhge	0,049834	0,258872

Table S3: Single features significant at the univariate analysis for 2yDFS prediction.

	features.name_def	all.pvalues	padj
1	ypN	0,000398	0,128101
2	pre_GTV_F_morph.pca.flatness	0,000436	0,128101
3	Stadio patologico	0,001273	0,249072
4	post_Mesoretto_F_cm.clust.shade	0,002892	0,424443
5	ycT	0,005869	0,675997
6	delta_GTV_L_least	0,00691	0,675997
7	post_GTV_F_rlm.rlnu	0,013186	0,926221
8	delta_GTV_L_minor	0,014613	0,926221
9	post_GTV_L_minor	0,015531	0,926221
10	post_GTV_F_szm.zsnu	0,017871	0,926221
11	ycM	0,021775	0,926221
12	post_GTV_F_szm.z.entr	0,023921	0,926221
13	post_GTV_F_stat.energy	0,02679	0,926221
14	post_GTV_F_morph.surface	0,027295	0,926221
15	post_GTV_F_morph.volume	0,030501	0,926221
16	post_GTV_F_cm.corr	0,031064	0,926221
17	pre_Mesoretto_F_morph.pca.flatness	0,031637	0,926221
18	post_GTV_F_cm.joint.entr	0,032809	0,926221
19	MRF.y	0,033095	0,926221
20	post_GTV_F_cm.energy	0,035263	0,926221
21	post_GTV_F_cm.sum.entr	0,0359	0,926221
22	post_GTV_F_rlm.rl.entr	0,0359	0,926221
23	post_GTV_L_least	0,037203	0,926221
24	post_GTV_F_rlm.glnu.norm	0,037869	0,926221
25	post_GTV_F_morph.av	0,040635	0,930876
26	post_Mesoretto_F_cm.sum.entr	0,042079	0,930876
27	post_GTV_F_szm.glnu.norm	0,042817	0,930876
